# Deep Brain Stimulation of the subthalamic nucleus does not negatively affect social cognitive abilities of patients with Parkinson’s disease

**DOI:** 10.1038/s41598-017-09737-6

**Published:** 2017-08-25

**Authors:** Ivan Enrici, Antonia Mitkova, Lorys Castelli, Michele Lanotte, Leonardo Lopiano, Mauro Adenzato

**Affiliations:** 1 Department of Philosophy and Educational Sciences, University of Turin, Turin, Italy; 2 Center for Cognitive Science, University of Turin, Turin, Italy; 3 Neuroscience Institute of Turin, Turin, Italy; 4 Psychology Research Laboratory, IRCCS Istituto Auxologico Italiano, Ospedale S. Giuseppe, Piancavallo, VCO Italy; 5 Department of Psychology, University of Turin, Turin, Italy; 6 Department of Neuroscience, University of Turin, Turin, Italy

## Abstract

Bilateral deep brain stimulation (DBS) of the subthalamic nucleus (STN) is a treatment option for patients with advanced idiopathic PD successful at alleviating disabling motor symptoms. Nevertheless, the effects of STN-DBS on cognitive functions remain controversial and few studies have investigated modification of social cognitive abilities in patients with PD treated with STN-DBS. Here we expanded the typically-investigated spectrum of these abilities by simultaneously examining emotion recognition, and both affective and cognitive Theory of Mind (ToM). By means of a cross-sectional study, 20 patients with PD under dopaminergic replacement therapy, 18 patients with PD treated with STN-DBS, and 20 healthy controls performed the Ekman 60-Faces test, the full version of the Reading the Mind in the Eyes test, and the Protocol for the Attribution of Communicative Intentions. There were no differences between the PD groups (treated and not treated with STN-DBS) on any of the social cognitive tests. Our results suggest that patients with PD who are treated with STN-DBS do not experience detrimental effects on their social cognitive abilities. The present study, the first one examining a wide spectrum of social cognitive abilities after DBS of the STN, suggests that this surgical procedure can be considered safe from this standpoint.

## Introduction

Parkinson’s Disease (PD) is a neurodegenerative disease associated with clinical symptoms that include bradykinesia, rigidity, resting tremor, and postural instability. Although PD is characterized primarily by motor symptoms, cognitive impairment and psychiatric disorders can be also observed even in the earliest stages of the disease^1, 2^. Cognitive deficits in patients with PD result from dopamine depletion and are attributed to disruption of the reciprocal loops between the striatum and structures in the prefrontal cortex. Therefore, individuals with PD may experience impairments in various cognitive domains that primarily depend on the frontal lobes. Furthermore, patients with PD have impaired performance in social cognitive tasks, such as recognition of emotional facial expression and those requiring Theory of Mind (ToM), i.e. the ability to explain and predict other people’s mental states^3–5^.

Chronic bilateral deep brain stimulation (DBS) of the subthalamic nucleus (STN) is a treatment option for patients with advanced idiopathic PD. STN-DBS is specifically indicated in patients for whom long-term pharmacological treatment has failed, and is remarkably successful at alleviating disabling motor symptoms^6^. Nevertheless, the effects of STN-DBS on cognitive functions, mood, and behaviour remain controversial, and experimental findings in this domain are sometimes contradictory^7–11^. Although cognitive difficulties such as moderate declines in executive functions, verbal memory, and fluency tasks have been reported, studies with large patient cohorts indicate that STN-DBS does not produce general cognitive declines^12, 13^. However, clinical and psychiatric issues, such as depression^8, 14^ and (hypo)mania^15^ have been reported following STN-DBS. Furthermore, DBS may also result in social maladjustment and aggravation of other pre-existing psychiatric disorders^16, 17^.

Previous research has examined emotional processing following STN-DBS, focusing primarily on subjective emotions and arousal, as well as emotion recognition^17^ (see Mallet *et al*., 2007 for a review). The effect of STN-DBS on emotion processing and its reported effects on cognition and mood are likely related to the role of the STN in the basal ganglia network. The DBS modifies dopaminergic transmission in the basal ganglia, affecting the limbic, associative, and motor networks via highly organised circuits^18, 19^. These basal ganglia circuits are comprised of both cortical (orbitofrontal cortex, temporal sulcus, temporal poles, cingulate gyrus and prefrontal cortex) and subcortical regions (amygdala and ventral striatum), and correspond with structures that also mediate social cognition^20, 21^. Furthermore, the basal ganglia networks also directly target the prefrontal cortex, including the dorsolateral prefrontal cortex and the anterior cingulate cortex.

Several studies have investigated social cognitive domains in patients with PD, whereas very few studies have tested modification of these domains by directly comparing patients with PD who are either treated or not treated with STN-DBS. In particular, no study has simultaneously investigated the effects of STN-DBS on (1) emotion recognition, i.e. reading facial expressions of emotion, (2) affective ToM (emotion representation), i.e., the ability to represent others’ emotions, and (3) cognitive ToM, i.e. the ability to represent others’ beliefs and intentions^22, 23^.

The aim of the present study was to examine the effects of STN-DBS on social cognitive abilities, particularly on emotion recognition, affective ToM, and cognitive ToM. This study aim at expanding knowledge regarding social cognitive abilities by testing patients’ abilities in both emotion recognition and representation, as well as by assessing both affective ToM and cognitive ToM. The rationale for investigating these features was to increase understanding regarding the complex clinical outcomes following STN-DBS, especially in relation to the non-motor symptoms of PD.

## Results

### Cognitive functioning assessment and demographic measures

The cognitive functioning assessment and demographic data for the DRT-PD, STN-DBS-PD, and HC groups are presented in Table 1. Experimental groups were matched for demographic variables such as age, education, and gender. Regarding the cognitive functioning assessment, experimental groups did not differ based on MMSE scores, whereas significant group differences occurred for the FAB scores (H_(2)_ = 8.221, p = 0.016). Pairwise Mann–Whitney tests revealed significant differences between DRT-PD and STN-DBS-PD (U = 103.5, p = 0.023, *η* ^2^ = 0.132) groups, as well as STN-DBS-PD and HC (U = 267.5, p = 0.009, *η* ^2^ = 0.172) groups, with participants in the STN-DBS-PD group performing worse than participants in the DRT-PD and HC groups.

**Table 1:** Cognitive functioning assessment and demographic measures.

	DRT-PD n = 20	STN-DBS-PD n = 18	HC n = 20
Age (years)	59.75 (5.81) (range = 50–70)	60.89 (6.26) (range = 41–70)	60 (7.47) (range = 45–71)
Education (years)	7.65 (2.52)	7.44 (3.85)	9.35 (3.34)
Gender (M:F)	10:10	9:9	10:10
MMSE	28.75 (1.04)	28.86 (1.39)	29.27 (1.06)
FAB	17.13 (1.12)	**15. 94 (1.66)** ^**a,b**^	17.34 (0.80)

Significant group effects found (a) between STN-DBS-PD vs. DRT-PD groups and (b) between STN-DBS-PD vs. HC groups. DRT-PD = patients with PD receiving dopaminergic replacement therapy; FAB = Frontal Assessment Battery; HC = healthy controls; MMSE = Mini Mental State Examination; PD = Parkinson’s disease; STN-DBS PD = patients with PD who were treated with deep brain stimulation of the subthalamic nucleus (STN-DBS).

### Social cognitive assessment

The difference in emotion recognition performance between DRT-PD, STN-DBS-PD and HC groups (i.e., Ekman total score, as well as the basic emotions of sadness, happiness, anger, disgust, fear, and surprise), affective ToM, and cognitive ToM are presented in Table 2. Significant group effects occurred for the Ekman 60-Faces total score (H_(2)_ = 6.824, p = 0.033), and for anger (H_(2)_ = 7.094, p = 0.029) and surprise (H_(2)_ = 11.129, p = 0.004), but not for sadness, happiness, disgust, or fear. Moreover, significant group differences occurred for both the CInt (H_(2)_ = 19.469, p < 0.001) and PhC (H_(2)_ = 7.275, p = 0.026) conditions of the PACI, and for the RME experimental score (H_(2)_ = 11.861, p = 0.003), but not for the RME control score.

**Table 2:** Social cognitive measures.

	Maximum score	DRT-PD n = 20	STN-DBS-PD n = 18	HC n = 20
Emotion recognition				
Ekman 60-Faces total score	60	43 (5.93)	43.89 (6.40)	48.15 (6.74)
Ekman-sadness	10	6.45 (1.96)	6.33 (1.64)	7.2 (1.70)
Ekman-happiness	10	9.65 (0.81)	9.56 (0.78)	9.65 (0.67)
Ekman-anger	10	6.15 (2.11)	7.56 (1.29)	7.75 (1.86)
Ekman-disgust	10	8 (1.95)	7.89 (1.71)	8.3 (2.03)
Ekman-fear	10	4.30 (2.30)	4.33 (2.77)	5.3 (2.88)
Ekman-surprise	10	8.45 (1.57)	8.22 (1.93)	**9.6 (0.82)** ^**a**^
Cognitive ToM (PACI task)				
Communicative intention	9	6.15 (1.93)	6.06 (2.3)	**8.3 (1.08)** ^**a,b**^
Physical causality (control)	9	7.10 (2)	5.94 (2.9)	7.95 (1.67)
Affective ToM				
RME	36	19.65 (4.44)	19.44 (4.87)	**24.15 (4.65)** ^**a,b**^
RME (control)	36	34.60 (1.23)	34.39 (1.65)	34.65 (1.39)

Significant group effects found (a) between STN-DBS-PD vs. HC groups, and (b) between DRT-PD vs. HC groups. DRT-PD = patients with PD receiving dopaminergic replacement therapy; HC = healthy controls; PACI = Protocol for the Attribution of Communicative Intentions; PD = Parkinson’s disease; RME = Reading the Mind in the Eyes Test; ToM = Theory of Mind; STN-DBS PD = patients with PD who were treated with deep brain stimulation of the subthalamic nucleus (STN-DBS).

Pairwise Mann–Whitney tests revealed no statistically significant differences between the DRT-PD and STN-DBS-PD groups for social cognitive dimensions, with the exception of anger (U = 250, p = 0.041), for which the STN-DBS-PD group performed better than the DRT-PD group. However, this difference disappeared after correction for multiple comparisons, suggesting a spurious effect.

Pairwise Mann–Whitney tests corrected for multiple comparisons revealed significant differences between the DRT-PD and HC groups for the CInt condition (U = 347, p < 0.001, *η* ^2^ = 0.395), and the RME experimental score (U = 315, p = 0.001, *η* ^2^ = 0.242). There were also significant differences between the STN-DBS-PD and HC groups for Ekman-surprise (U = 273.5, p = 0.005, *η* ^2^ = 0.197), CInt condition (U = 297, p < 0.001, *η* ^2^ = 0.308), and RME experimental scores (U = 275, p = 0.005, *η* ^2^ = 0.203).

In order to account for the possible effects of FAB performance on social cognitive measures, we performed further analyses using a non-parametric analysis of covariance, Quade’s rank analysis^24^. After statistically controlling for the FAB scores, the previously described significant group effects persisted.

The differences in performance between the two groups of patients with PD for the neuropsychological, psychiatric and, clinical measures are presented in Tables 3 and 4. No significant group effects occurred for the neuropsychological measures, with the exception of the phonemic verbal fluency score (U = 66, p = 0.001, *η* ^2^ = 0.292), for which patients in the DRT-PD group performed better than patients in the STN-DBS-PD group.

**Table 3:** Neuropsychological measures.

	DRT-PD n = 20	STN-DBS-PD n = 18	Mann-Whitney U-test
Corsi’s Block-Tapping Test	4.76 (0.67)	4.58 (0.48)	U = 163 p = 0.633
Attentional matrices	47.01 (7.31)	43.11 (7.65)	U = 132.5 p = 0.167
Raven’s Coloured Progressive Matrices	30.51 (4.34)	31.53 (3.44)	U = 202.5 p = 0.515
Bisyllabic word repetition test	4.13 (0.71)	4.06 (0.94)	U = 182.5 p = 0.942
Paired-associate learning	13.24 (2.91)	12.09 (3.51)	U = 132.5 p = 0.363
Trail Making Test, Part B	107.20 (136.98)	120.56 (82.29)	U = 232 p = 0.133
NCST - categories	5.80 (0.41)	5.89 (0.32)	U = 196 p = 0.654
NCST - errors	5.25 (3.19)	3.50 (2.64)	U = 122.5 p = 0.093
NCST - perseverations	2.40 (1.85)	2.00 (1.88)	U = 163 p = 0.633
Phonemic verbal fluency	39.95 (7.72)	31.67 (6.02)	**U = 66 p = 0.001**
Category verbal fluency	21.64 (3.18)	22.83 (15.97)	U = 108.5 p = 0.057

DRT-PD = patients with PD receiving dopaminergic replacement therapy; HC = healthy controls; NCST = Nelson Modified Card Sorting Test; PD = Parkinson’s disease; RME = Reading the Mind in the Eyes Test; STN-DBS-PD = patients with PD who were treated with deep brain stimulation of the subthalamic nucleus (STN-DBS).

**Table 4:** Psychiatric and clinical measures.

	DRT-PD n = 20	STN-DBS-PD n = 18	Mann-Whitney U-test
BDI	13.05 (6.66)	11.61 (6.05)	U = 146.5 p = 0.461
STAI-X1	46.47 (10.42)	41.22 (10.00)	U = 125 p = 0.169
STAI-X2	44.95 (10.63)	41.61 (8.43)	U = 134.5 p = 0.271
Apathy Evaluation Scale	12.84 (5.56)	11.88 (3.59)	U = 145 p = 0.616
LEDD	1074.45 (431.60)	760.44 (384.29)	**U = 102 p = 0.036**
LEDD-Dopa	973.13 (427.15)	735.39 (356.64)	U = 113.5 p = 0.081
LEDD-DA	100.84 (98.10)	47.28 (55.18)	U = 115 p = 0.092
H&Y Off	2.39 (0.53)	2.94 (0.70)	U = 200 p = 0.055
H&Y On	1.42 (0.58)	2.06 (1.08)	U = 193.5 p = 0.088
UPDRS III Off	33.13 (8.83)	39.53 (13.77)	U = 198 p = 0.133
UPDRS III On	12.18 (4.58)	12.53 (6.46)	U = 155 p = 0.935
Duration of illness (years)	11.50 (2.98) (range = 8–20)	12.56 (3.03) (range = 10–20)	U = 218 p = 0.276

BDI = Beck Depression Inventory; DRT-PD = patients with PD receiving dopaminergic replacement therapy; H&Y = Hoehn and Yahr scale; LEDD = L-dopa equivalent daily dose; LEDD-Dopa = LEDD-dopaminergic portion; LEDD-DA = LEDD-dopamine agonists; PD = Parkinson’s disease; STAI = State Trait Anxiety Inventory; STN-DBS-PD = patients with PD who were treated with deep brain stimulation of the subthalamic nucleus (STN-DBS); UPDRS = Unified Parkinson’s Disease Rating Scale.

Furthermore, the patient groups were well-matched based on the clinical variables, with the exceptions of the LEDD (U = 102, p = 0.036, *η* ^2^ = 0.137), for which patients in the DRT-PD group took a higher dosage compared to patients in the STN-DBS-PD group.

In order to investigate the possible relationships between social cognitive (Ekman 60-Faces, PACI, and RME) and psychiatric (BDI, STAI, and Apathy Evaluation Scale) measures, we analysed bivariate correlations in the two groups of patients with PD, between each dimension (see Table 5 in the supplementary materials). For the DRT-PD group, there were significant correlations only between STAI measures and the CInt condition (STAI-X1, τb = −0.559, p = 0.002 and STAI-X2, τb = −0.517, p = 0.004), between STAI-X1 and the PhC condition (τb = −0.417, p = 0.020) and between BDI measures and the CInt condition (τb = −0.361, p = 0.044). For the STN-DBS-PD group, there were significant correlations between STAI-X1 and the CInt condition (τb = −0.506, p = 0.005), RME (τb = −0.440, p = 0.014), and surprise (τb = −0.391, p = 0.035), as well as between STAI-X2 and Ekman total scores (τb = −0.401, p = 0.022) and surprise (τb = −0.432, p = 0.019). Lastly, significant correlations occurred between the Apathy Evaluation Scale scores and Ekman total scores (τb = −0.397, p = 0.033), as well as between the Apathy Evaluation Scale scores and happiness (τb = −0.417, p = 0.047).

In addition, in the two groups of patients with PD, we investigated possible correlations between social cognitive measures (Ekman 60-Faces, PACI, and RME), dopaminergic pharmacological treatment (LEDD), duration of illness, and severity of the disease (UPDRS III Off) (see Table 5 in the supplementary materials). There were no statistically significant correlations, except for correlations between sadness and duration of illness (τb = −0.401, p = 0.041), and between anger and UPDRS III Off scores (τb = −0.420, p = 0.036) in the STN-DBS-PD group, as well as between UPDRS III Off scores and CInt (τb = 0.434, p = 0.014), and PhC (τb = 0.455, p = 0.011) in the DRT-PD group.

Finally, for exploratory purpose, we performed bivariate correlations between social cognitive and neuropsychological measures in the two groups of patients with PD (see Tables 6 and 7 in the Supplementary Materials).

## Discussion

The main aim of the present paper was to investigate the effects of STN-DBS on the social cognitive abilities of patients with PD. Our results strongly suggest that STN-DBS does not negatively affect social cognitive abilities of patients with PD. We expanded the typically investigated spectrum of social cognitive abilities by investigating emotion recognition, affective ToM (emotion representation), and cognitive ToM. There were no differences between the PD groups on any of the social cognitive tasks. DRT-PD, STN-DBS-PD and HC groups were matched for age, gender, and education, as well as for general cognitive functioning, as assessed by MMSE scores. In addition, the two PD groups were well-matched for duration of illness, and severity of the disease. No significant differences between the two PD groups occurred for psychiatric (depression, anxiety, and apathy) or neuropsychological scales, with the exception of phonemic fluency. The phonemic fluency score indicated impaired ability in the STN-DBS-PD group compared to the DRT-PD and HC groups, which is a well-documented outcome of STN-DBS^13^. There was no correlation between social cognition tasks and LEDD.

### Emotion recognition

Our results show that STN-DBS, when combined with optimal medical treatment, does not result in impairment of emotion recognition in patients with PD. We found no differences between the PD groups nor on Ekman 60-Faces total score nor on any of Ekman basic emotion, whereas only Ekman-surprise differed between the STN-DBS-PD and HC groups, with participants in the STN-DBS-PD group performing worse than participants in the HC groups.

Previous research suggests that patients with PD experience impaired facial emotion expression recognition; however, the results are inconclusive regarding the degree and selectivity of emotion recognition impairments^25^. Impaired recognition of disgust has been described in both un-medicated and medicated patients with PD^26^. Other authors have investigated the relationships with neuropsychiatric and neuropsychological symptoms, again yielding mainly inconclusive results^27^.

The effects of STN-DBS on recognition of facial emotion expressions have yielded contrasting findings, likely due to different study methods. However, the overall results suggest that STN-DBS may affect the ability to recognise negative facial expressions, in particular anger^28^, and disgust^29, 30^, as well as fear and sadness^31–33^. In contrast, Mondillon, *et al*.^34^ found no impairment in emotion recognition in patients with STN-DBS-PD who were evaluated while receiving both dopaminergic and DBS therapies (Stim On-Med On condition). Moreover, Albuquerque, *et al*.^35^ reported no differences in emotion recognition in patients with advanced PD after STN-DBS, based on comparisons of the ability to recognize emotions before and one year after surgery. It is important to note that previous studies have administered an emotion recognition task that uses pictures of the Ekman 60-Faces Test^31–33, 36^, showing the pictures for 3 sec. In the present study, we chose not to use a specified presentation duration, in order to reduce the demand on attention and working memory. Moreover, our version of the Ekman 60-Faces Test is comprised of 10 stimuli for each basic emotion, whereas previous studies have used a modified version of the test that consists of fewer stimuli for each emotion.

Our results confirm the findings by Mondillon, *et al*.^34^, who found no impairment of facial expression recognition in patients with STN-DBS-PD in an optimal medication and stimulation condition, compared to an HC group. The authors of this study did not assess the patients for facial expression recognition pre-STN-DBS and did not have a control group of patients with PD, therefore, we cannot make comparisons for all aspects of the study.

### Affective ToM

Our findings show that patients with PD who are treated with STN-DBS do not experience detrimental effects on affective ToM. No differences between the PD groups on the RME were found, whereas the performance on the RME differed between both the PD groups and HC, with participants in the STN-DBS-PD and DRT-PD groups performing worse than participants in the HC group.

Consistent with most previous studies, we observed impairments in emotion representation in patients with PD. We have previously suggested that the decline in this ability depends on the spatiotemporal progression of dopamine depletion in patients with PD, indicating that emotion representation abilities may be impaired at more advanced stages of PD^5, 25, 37^. These declines may be mediated by dopaminergic depletion-induced hypostimulation of the ventromedial prefrontal cortex by the frontostriatal loops, since the ventromedial prefrontal cortex contributes to emotion representation.

Two previous studies have assessed affective ToM in patients with STN-DBS-PD, using the RME^38, 39^, however, these studies yielded contrasting results. McIntosh, *et al*.^39^ administered the RME to patients with early PD, who were randomised to receive either DRT (n = 7) or STN-DBS surgery (n = 9). Due to small sample size, the authors did not compare the two groups directly and did not suggest any conclusions regarding the comparison. Investigating the effect of treatment type (DRT vs. STN-DBS) and treatment state (ON/OFF) tasks, these authors found no significant effects of therapy on RME performance. Peron, *et al*.^38^ correlated RME performance to changes in glucose metabolism by conducting fludeoxyglucose positron emission tomography scans of 13 patients with PD at both three months prior to and three months post-STN-DBS surgery. The authors examined RME performance using a reduced 17 item version of the test, and compared the performance of the PD group pre- and post-surgery with that of an HC group (n = 13). They found no significant difference between preoperative PD and HC results, whereas postoperative patients performed significantly worse than either preoperative PD or HC groups.

The discrepancies between our results and those of Peron, *et al*.^38^ may have resulted from different study methods. As previously discussed, most studies that have used the RME in patients with PD have found impaired emotion representation. Peron, *et al*.^38^ found that the pre-surgery performance of patients with PD did not differ from that of participants in the HC group, indicating that these abilities were preserved in their PD group. Furthermore, there are methodological differences regarding the versions of the RME administered, with Peron, *et al*.^38^ using a shorter 17 item test, whereas we used the full version comprised of 36 items. It has been suggested that using short versions of the RME is problematic, as mild or sub-threshold impairments in affective ToM may not be detected^25, 37^.

### Cognitive ToM

The present study is the first examination of cognitive components of ToM after DBS of the STN in patients with PD, and our results show that STN-DBS has no detrimental effects on cognitive ToM. No differences between the PD groups on PACI tasks were found, whereas the performance on CInt differed between both the PD groups and HC, with participants in the STN-DBS-PD and DRT-PD groups performing worse than participants in the HC group.

The experimental protocol that we used to study intention attribution was initially developed for a series of fMRI studies aimed at exploring the theoretical classification of intentions^40–45^. The results of these studies strongly implicate the prefrontal cortices in the ability to understand the intentions of people involved in communicative interactions (i.e. cognitive ToM). The prefrontal cortices are critical to the neuropathology of PD, as well as in STN networks, and are also implicated in cognitive ToM; therefore, effects of STN-DBS on this latter ability cannot be excluded. Furthermore, numerous reports support impairment of cognitive ToM in patients with PD^37, 46^, leading to the question of whether an existing impairment changes as a result of stimulation. Thus, the present findings corroborate the hypothesis that tasks requiring the comprehension of others’ intention may be impaired in both PD groups and, more importantly, suggest that STN-DBS has no detrimental effects on cognitive ToM.

## Conclusion

STN-DBS surgery is commonly used to treat the motor symptoms of patients with PD. Research has begun to investigate the effects of STN-DBS on mood and cognition, and factors such as subtle differences in electrode placement, stimulation parameters, patient vulnerability prior to STN-DBS, and dopamine dose changes may influence the outcomes of STN-DBS in patients with PD. Since non-motor effects of STN-DBS have the potential for large impacts on patient quality of life, it is essential to assess possible effects of STN-DBS on every aspect of cognition.

STN-DBS may also provide a useful model for studying the role of the STN in emotional processes and ToM^20, 38^. Most previous studies focus on emotion recognition following STN-DBS, however, the present study investigated different facets of social cognition, namely emotion recognition, affective ToM, and cognitive ToM.

The present study has two main limitations. Firstly, the sample size is relatively small, and future studies should aim to replicate our findings using larger samples. Secondly, we used only the MMSE and FAB as neuropsychological measures to compare the two groups of PD with the HC group. Despite these limitations, the present study is the first investigation of the effects of STN-DBS on social cognition, using measures to assess emotion recognition and representation, as well as both cognitive and affective ToM. We found no specific effects of STN-DBS on emotion recognition, cognitive ToM and affective ToM. The results of the present study contribute to knowledge regarding the cognitive safety of STN-DBS treatment. Our results support that STN-DBS is safe in relation to the social cognitive domain and does not have detrimental effects on the social cognitive abilities investigated.

## Materials and Methods

### Ethics statement

Written informed consent was obtained from all participants. The study was approved by the San Giovanni Battista University Hospital’s ethics committee and was conducted in accordance with the Declaration of Helsinki.

### Participants

By means of a cross-sectional study, 20 consecutive patients with PD under dopaminergic replacement therapy (DRT), 18 patients with PD treated with STN-DBS, and 20 healthy controls (HC) with negative neurological and psychiatric history were enrolled in the study. All patients were consecutively referred to the neurology unit of Turin University Hospital for standard care visits. Inclusion criteria included a diagnosis of idiopathic PD, and age ≥40 years. Exclusion criteria for all groups of participants were the presence of dementia or severe cognitive impairment (Mini Mental State Examination, MMSE ≤ 26), and presence of other neurological or psychiatric disorders, such as severe depression as assessed using a psychological interview and the Beck Depression Inventory (BDI)^47^, which is a validated measure for depression symptoms in patients with PD^48^.

All patients received their daily optimal dopamine replacement therapies (L-dopa preparations, and/or dopamine receptor agonists).

### Motor evaluations

Motor evaluations were performed according to the Unified Parkinson’s Disease Rating Scale, Part III (UPDRS-III)^49^. Scores were measured at baseline (after overnight withdrawal of all dopaminergic medication) and after administration of a loading dose of L-dopa (calculated as 1.5× the usual L-dopa equivalent morning dose).

In the first clinical group (DRT-PD), UPDRS scores were measured during two conditions: on-medication (Med On) and off-medication (Med Off). The second group (STN-DBS-PD) was assessed during four different conditions: with stimulation switched on after overnight medication withdrawal (Stim On-Med Off); 60 min after switching off stimulation, after overnight medication withdrawal (Stim Off-Med Off); with stimulation switched off and about 40 minutes after the administration of L-dopa therapy, at the same dose used for the preoperative test (Stim Off-Med On); and undergoing both stimulation and L-dopa therapy condition (Stim On-Med On). In the present study we analysed the UPDRS Off conditions, i.e., the Med Off condition for DRT-PD group and Stim Off-Med Off for STN-DBS-PD, in order to compare the severity of the disease between the two PD groups. Furthermore, Hoehn and Yahr (H&Y)^50^ scale was administered in order to assess the progression of motor symptoms in both On and Off conditions. L-dopa equivalent daily doses (LEDD) were calculated according to commonly established standard conversions^51^.

### Neurosurgery

The surgical procedure^52^ involved bilateral electrode implantation in the STN, which was accomplished during two independent surgeries. Anatomical targeting was performed using magnetic resonance imaging (MRI)/computed tomography image fusion. During surgery, clinical effects were evaluated using electro-physiological recordings and microstimulation, in order to achieve the best possible lead placement. Postoperative MRI was performed in order to confirm electrode positioning and to exclude surgical complications.

Eligibility criteria for DBS included diagnosis of idiopathic PD, presence of severe motor fluctuations and/or drug-related dyskinesias, and clinical response to L-dopa treatment for motor symptoms. Moreover, patients were <70 years of age, did not have dementia and/or relevant psychiatric comorbidities, and did not have relevant atrophy or focal abnormalities on brain MRI. The patient selection was performed based on the Core Assessment Program for Surgical Interventional Therapies in Parkinson’s Disease (CAPSIT-PD)^53^.

### Background neuropsychological and psychiatric assessments

The MMSE^54^ and the Frontal Assessment Battery (FAB)^55, 56^ were administered to all participants prior to social cognitive tasks, as a preliminary screening for global cognitive and executive functioning. In addition, a standardised neuropsychological battery of tests^10^ was administered to both DRT-PD and STN-DBS-PD groups, in order to evaluate reasoning, memory, attention, and executive functions. Reasoning was assessed using Raven’s Coloured Progressive Matrices^57^. Short term memory for verbal (bi-syllabic word repetition test) and visuospatial components (Corsi’s Block Tapping Test)^58^ were assessed, and long term memory was assessed using the Wechsler memory scale subtest, which is a paired-associate learning test^59^. Attention and executive functions were assessed using attentional matrices^58^, the Trail Making Test, Part B^60^, the Nelson Modified Card Sorting test^61, 62^, as well as phonemic^58^ and category verbal fluency tasks^59^.

Psychiatric assessments included evaluation of apathy anxiety and depression using the Apathy Evaluation Scale^63^, the State Trait Anxiety Inventory (STAI)^64^, in particular (STAI-X1) and (STAI-X2), and the BDI^47^.

### Social cognition tasks

The DRT-PD, STN-DBS-PD, and HC groups performed three social cognitive tasks: the Ekman 60-Faces Test for emotion recognition, the 36-item full version of the Reading the Mind in the Eyes (RME) test for affective ToM, and the Protocol for the Attribution of Communicative Intentions (PACI) for cognitive ToM.

The ability to recognise basic facial emotions was evaluated using the Italian version of the Ekman 60-Faces Test^65, 66^. This task assesses both overall emotion recognition and basic emotion detection. It consists of 60 black and white pictures that portray the faces of 10 actors, each displaying the six basic emotions (happiness, surprise, anger, disgust, fear, and sadness). A computerised sequence of slides containing a facial emotion picture was administered to participants, requiring them to select the label that best described the facial expression in the picture. The labels were visible throughout testing and participants were allowed as much time as they needed to verbally make their selection. No feedback was given throughout testing.

The RME^67^ is an advanced test of affective ToM (emotion representation) that consists of 36 partial pictures of a face depicting only the eye region. The task was presented on a computer screen. For each photograph, participants were asked to choose one of four words that best described what the character was thinking or feeling. One correct and three distractor words were presented for each item. Explanations of the labels were given when requested by participants, using the definitions provided by the Italian validation of the task^68^. The score was calculated by counting the total number of correct choices (maximum score 36). In order to exclude visual impairment, we administered the RME gender-recognition control task, which required participants to identify the gender of the character in the photograph.

The PACI is an intention attribution task^40, 42, 69, 70^ that distinguishes between social and physical interactions, and was administered as a measure of cognitive ToM. A series of 18 comic strips was presented to participants, representing two different conceptual categories: communicative intentions (CInt), and physical causality (PhC). The CInt condition consisted of nine stories that each portrayed two characters involved in a communicative interaction (e.g. a person asking for a glass of water and obtaining it from another person). The PhC condition consisted of nine stories that each depicted a physical interaction between objects (e.g. a ball blown by a gust of wind knocks over and breaks bottles of water), and did not require ToM abilities. Each story consisted of three pictures (development phase) that appeared consecutively on the screen, followed by a set of four options (response phase), and participants selected the most appropriate and logical story ending. The correct picture represented a probable and congruent outcome resulting from the development phase, while the incorrect pictures represented an improbable or incongruent effect. In order to avoid working memory overload and inaccuracies due to slowed information processing speed, patients were allowed an unlimited duration of time to respond, and all pictures remained on the screen while participants completed the story. Examples of the stories can be found at the following web address: www.psych.unito.it/csc/pers/adenzato/pdf/stn_dbs_pd.pdf.

### Procedure

The neuropsychological assessment and the experimental tasks were performed under optimal clinical conditions for all participants. In particular, patients in the DRT-PD group, who were not treated with STN-DBS, were assessed in the Med On condition, while patients in the STN-DBS group were assessed in the Stim On-Med On condition. Patients in the STN-DBS-PD group were evaluated at least six months after surgery, in order to minimise microlesion effects^71^, with a mean follow up of 1.72 (±1.18) years.

### Statistical analyses

Statistical analyses were conducted using SPSS version 24.0 for Windows. There were a limited number of participants in each group and significant deviations from normality of variable distribution, as assessed by Shapiro-Wilk’s tests (p < 0.05); therefore, non-parametric methods were used (Kruskal-Wallis H-test, Mann-Whitney U-test, and Kendall’s tau-b correlation coefficient). Comparisons of the three independent groups were performed using the Kruskal-Wallis H-test. When the Kruskal–Wallis test yielded a significant difference, pairwise Mann–Whitney tests were conducted in order to determine specific differences with a significance level of 0.05, Bonferroni-corrected for multiple comparisons. The effect size was determined by calculating Eta squared (*η* ^2^).

## Electronic supplementary material

Tables 5, 6 and 7

## References

[1] Hirano S, Shinotoh H, Eidelberg D (2012). Functional brain imaging of cognitive dysfunction in Parkinson’s disease. J Neurol Neurosur Ps.

[2] Aarsland D, Bronnick K, Fladby T (2011). Mild Cognitive Impairment in Parkinson’s Disease. Curr Neurol Neurosci.

[3] Bodden ME, Dodel R, Kalbe E (2010). Theory of Mind in Parkinson’s Disease and Related Basal Ganglia Disorders: A Systematic Review. Movement Disord.

[4] Freedman M, Stuss DT (2011). Theory of Mind in Parkinson’s disease. J Neurol Sci.

[5] Poletti M, Enrici I, Bonuccelli U, Adenzato M (2011). Theory of Mind in Parkinson’s disease. Behav Brain Res.

[6] Benabid AL, Chabardes S, Mitrofanis J, Pollak P (2009). Deep brain stimulation of the subthalamic nucleus for the treatment of Parkinson’s disease. Lancet Neurol.

[7] Wu B, Han L, Sun BM, Hu XW, Wang XP (2014). Influence of deep brain stimulation of the subthalamic nucleus on cognitive function in patients with Parkinson’s disease. Neurosci Bull.

[8] Castrioto A, Lhommee E, Moro E, Krack P (2014). Mood and behavioural effects of subthalamic stimulation in Parkinson’s disease. Lancet Neurol.

[9] Halpern CH, Rick JH, Danish SF, Grossman M, Baltuch GH (2009). Cognition following bilateral deep brain stimulation surgery of the subthalamic nucleus for Parkinson’s disease. Int J Geriatr Psychiatry.

[10] Castelli L (2010). Neuropsychological changes 1-year after subthalamic DBS in PD patients: A prospective controlled study. Parkinsonism Relat Disord.

[11] Castelli L (2008). Neuropsychiatric symptoms three years after subthalamic DBS in PD patients: a case-control study. J Neurol.

[12] Castelli L (2006). Chronic deep brain stimulation of the subthalamic nucleus for Parkinson’s disease: effects on cognition, mood, anxiety and personality traits. Eur Neurol.

[13] Parsons TD, Rogers SA, Braaten AJ, Woods SP, Troster AI (2006). Cognitive sequelae of subthalamic nucleus deep brain stimulation in Parkinson’s disease: a meta-analysis. Lancet Neurol.

[14] Gokbayrak NS, Piryatinsky I, Gavett RA, Ahmed OJ (2014). Mixed effects of deep brain stimulation on depressive symptomatology in Parkinson’s disease: a review of randomized clinical trials. Front Neurol.

[15] Temel Y (2006). Behavioural changes after bilateral subthalamic stimulation in advanced Parkinson disease: a systematic review. Parkinsonism Relat Disord.

[16] Houeto JL (2002). Behavioural disorders, Parkinson’s disease and subthalamic stimulation. J Neurol Neurosurg Psychiatry.

[17] Takeshita S (2005). Effect of subthalamic stimulation on mood state in Parkinson’s disease: evaluation of previous facts and problems. Neurosurg Rev.

[18] Mallet L (2007). Stimulation of subterritories of the subthalamic nucleus reveals its role in the integration of the emotional and motor aspects of behavior. P Natl Acad Sci USA.

[19] Temel Y, Blokland A, Steinbusch HWM, Visser-Vandewalle V (2005). The functional role of the subthalamic nucleus in cognitive and limbic circuits. Prog Neurobiol.

[20] Peron J, Fruhholz S, Verin M, Grandjean D (2013). Subthalamic nucleus: a key structure for emotional component synchronization in humans. Neurosci Biobehav Rev.

[21] Peron J (2015). Sensory contribution to vocal emotion deficit in Parkinson’s disease after subthalamic stimulation. Cortex.

[22] Mitchell RL, Phillips LH (2015). The overlapping relationship between emotion perception and theory of mind. Neuropsychologia.

[23] Di Tella M (2015). Theory of Mind and Emotional Functioning in Fibromyalgia Syndrome: An Investigation of the Relationship between Social Cognition and Executive Function. Plos One.

[24] Quade D (1967). Rank analysis of covariance. Journal of the American Statistical Association.

[25] Enrici, I. *et al*. Emotion Processing in Parkinson’s Disease: A Three-Level Study on Recognition, Representation, and Regulation. *Plos One* **10**, e0131470, doi:10.1371/journal.pone.0131470 (2015). 10.1371/journal.pone.0131470 PMC4482447 26110271

[26] Gray HM, Tickle-Degnen L (2010). A meta-analysis of performance on emotion recognition tasks in Parkinson’s disease. Neuropsychology.

[27] Assogna F, Pontieri FE, Caltagirone C, Spalletta G (2008). The recognition of facial emotion expressions in Parkinson’s disease. Eur Neuropsychopharmacol.

[28] Schroeder U (2004). Facial expression recognition and subthalamic nucleus stimulation. J Neurol Neurosurg Psychiatry.

[29] Dujardin K (2004). Subthalamic nucleus stimulation induces deficits in decoding emotional facial expressions in Parkinson’s disease. J Neurol Neurosurg Psychiatry.

[30] Aiello M (2014). Emotion recognition in Parkinson’s disease after subthalamic deep brain stimulation: differential effects of microlesion and STN stimulation. Cortex.

[31] Biseul I (2005). Fear recognition is impaired by subthalamic nucleus stimulation in Parkinson’s disease. Neuropsychologia.

[32] Peron J (2010). Subthalamic nucleus stimulation affects fear and sadness recognition in Parkinson’s disease. Neuropsychology.

[33] Drapier D (2008). Emotion recognition impairment and apathy after subthalamic nucleus stimulation in Parkinson’s disease have separate neural substrates. Neuropsychologia.

[34] Mondillon L (2012). The combined effect of subthalamic nuclei deep brain stimulation and L-dopa increases emotion recognition in Parkinson’s disease. Neuropsychologia.

[35] Albuquerque L, Coelho M, Martins M, Martins IP (2014). STN-DBS does not change emotion recognition in Parkinson’s disease. Parkinsonism Relat D.

[36] Le Jeune F (2008). Subthalamic nucleus stimulation affects orbitofrontal cortex in facial emotion recognition: a pet study. Brain.

[37] Poletti M, Enrici I, Adenzato M (2012). Cognitive and affective Theory of Mind in neurodegenerative diseases: Neuropsychological, neuroanatomical and neurochemical levels. Neurosci Biobehav R.

[38] Peron J (2010). Subthalamic nucleus stimulation affects theory of mind network: a PET study in Parkinson’s disease. Plos One.

[39] McIntosh, L. G. *et al*. Emotion recognition in early Parkinson’s disease patients undergoing deep brain stimulation or dopaminergic therapy: a comparison to healthy participants. *Front Aging Neurosci* **6**, doi:10.3389/fnagi.2014.00349 (2015). 10.3389/fnagi.2014.00349 PMC4301000 25653616

[40] Ciaramidaro A (2007). The intentional network: How the brain reads varieties of intentions. Neuropsychologia.

[41] Enrici I, Adenzato M, Cappa S, Bara BG, Tettamanti M (2011). Intention Processing in Communication: A Common Brain Network for Language and Gestures. J Cognitive Neurosci.

[42] Walter H (2004). Understanding intentions in social interaction: The role of the anterior paracingulate cortex. J Cognitive Neurosci.

[43] Walter H (2009). Dysfunction of the social brain in schizophrenia is modulated by intention type: an fMRI study. Soc Cogn Affect Neurosci.

[44] Tettamanti M (2017). Effective connectivity gateways to the Theory of Mind network in processing communicative intention. Neuroimage.

[45] Bara, B. G., Ciaramidaro, A., Walter, H. & Adenzato, M. Intentional minds: a philosophical analysis of intention tested through fMRI experiments involving people with schizophrenia, people with autism, and healthy individuals. *Front Hum Neurosci* **5**, doi:10.3389/fnhum.2011.00007 (2011). 10.3389/fnhum.2011.00007 PMC3034216 21344005

[46] Adenzato M, Poletti M (2013). Theory of Mind abilities in neurodegenerative diseases: An update and a call to introduce mentalizing tasks in standard neuropsychological assessments. Clinical Neuropsychiatry.

[47] Beck AT, Ward CH, Mendelson M, Mock J, Erbaugh J (1961). An inventory for measuring depression. Arch Gen Psychiatry.

[48] Leentjens AFG, Verhey FRJ, Luijckx GJ, Troost J (2000). The validity of the Beck Depression Inventory as a screening and diagnostic instrument for depression in patients with Parkinson’s disease. Movement Disord.

[49] Fahn, S. & Elton, R. L. In *Recent developments in Parkinson’s disease* Vol. 2 (eds S. Fahn, C. D. Marsden, D. B. Calne, & M. Goldstein) 153–164 (Macmillan Health Care Information, 1987).

[50] Hoehn MM, Yahr MD (1967). Parkinsonism: onset, progression and mortality. Neurology.

[51] Tomlinson CL (2010). Systematic Review of Levodopa Dose Equivalency Reporting in Parkinson’s Disease. Movement Disord.

[52] Lanotte MM (2002). Deep brain stimulation of the subthalamic nucleus: anatomical, neurophysiological, and outcome correlations with the effects of stimulation. J Neurol Neurosur Ps.

[53] Bronstein JM (2011). Deep Brain Stimulation for Parkinson Disease An Expert Consensus and Review of Key Issues. Arch Neurol-Chicago.

[54] Magni E, Binetti G, Bianchetti A, Rozzini R, Trabucchi M (1996). Mini-mental state examination: A normative study in Italian elderly population. Eur J Neurol.

[55] Dubois B, Slachevsky A, Litvan I, Pillon B (2000). The FAB - A frontal assessment battery at bedside. Neurology.

[56] Appollonio I (2005). The Frontal Assessment Battery (FAB): Normative values in an Italian population sample. Neurol Sci.

[57] Measso G (1993). Raven’s colored progressive matrices: a normative study of a random sample of healthy adults. Acta Neurol Scand.

[58] Spinnler, H. & Tognoni, G. Taratura e standardizzazione italiana di test neuropsicologici. *Italian Journal of Neurological Sciences* 1–120 (1987). 3330072

[59] Zappalà G (1995). Aging and memory: corrections for age, sex and education for three widely used memory tests. The Italian Journal of Neurological Sciences.

[60] Giovagnoli AR (1996). Trail making test: normative values from 287 normal adult controls. The Italian journal of neurological sciences.

[61] Caffarra P, Vezzadini G, Dieci F, Zonato F, Venneri A (2004). Modified Card Sorting Test: normative data. J Clin Exp Neuropsychol.

[62] Nelson HE (1976). A modified card sorting test sensitive to frontal lobe defects. Cortex.

[63] Marin RS, Biedrzycki RC, Firinciogullari S (1991). Reliability and validity of the Apathy Evaluation Scale. Psychiatry Res.

[64] Spielberger, C. D. Manual for the State-Trait Anxiety Inventory, Form Y (“self-evaluation questionnaire”). (Consulting Psychologists Press, 1983).

[65] Ekman P, Freisen WV, Ancoli S (1980). Facial signs of emotional experience. Journal of personality and social psychology.

[66] Dodich A (2014). Emotion recognition from facial expressions: a normative study of the Ekman 60-Faces Test in the Italian population. Neurol Sci.

[67] Baron‐Cohen S, Jolliffe T, Mortimore C, Robertson M (1997). Another advanced test of theory of mind: Evidence from very high functioning adults with autism or Asperger syndrome. Journal of Child Psychology and Psychiatry.

[68] Vellante M (2013). The “Reading the Mind in the Eyes” test: Systematic review of psychometric properties and a validation study in Italy. Cognitive Neuropsychiatry.

[69] Adenzato M (2017). Gender differences in cognitive Theory of Mind revealed by transcranial direct current stimulation on medial prefrontal cortex. Sci Rep.

[70] Cavallo M, Enrici I, Adenzato M (2011). The comprehension of social situations in a small group of patients with frontotemporal dementia and Alzheimer’s disease. Acta Neuropsychologica.

[71] Mann JM (2009). Brain penetration effects of microelectrodes and DBS leads in STN or GPi. J Neurol Neurosurg Psychiatry.

