# Supplementary material for: Deep Brain Stimulation of the subthalamic nucleus does not negatively affect social cognitive abilities of patients with Parkinson’s disease
Source: Sci Rep. 2017 Aug 25;7:9413. doi: 10.1038/s41598-017-09737-6 (PMC5573348; doi:10.1038/s41598-017-09737-6)
Supplement: Supplementary file 1 — Tables 5, 6 and 7 [file 41598_2017_9737_MOESM1_ESM.docx]

# Deep Brain Stimulation of the subthalamic nucleus does not negatively affect social cognitive abilities of patients with Parkinson's disease

Ivan Enrici§^1,2,3^, Antonia Mitkova§^4^, Lorys Castelli*^5^, Michele Lanotte^6,^

Leonardo Lopiano^6^, Mauro Adenzato^2,3,5^

§These authors contributed equally to this work

1. Department of Philosophy and Educational Sciences, University of Turin, Turin, Italy

2. Center for Cognitive Science, University of Turin, Turin, Italy

3. Neuroscience Institute of Turin, Turin, Italy

4. Psychology Research Laboratory, IRCCS Istituto Auxologico Italiano, Ospedale S. Giuseppe, Piancavallo (VCO), Italy

5. Department of Psychology, University of Turin, Turin, Italy

6. Department of Neuroscience, University of Turin, Turin, Italy

* Corresponding Author:

Lorys Castelli, Ph.D.

Department of Psychology, University of Turin, Turin, Italy

via Po 14, 10123 Turin, Italy

Phone: +39.011.670.3050

Email: lorys.castelli@unito.it

**Supplementary material**

**Table 5. Correlations between social cognitive, psychiatric and clinical measures in the two PD groups.**

|  | PD group | BDI | STAI-X1 | STAI-X2 | Apathy Evaluation Scale | Duration of illness | UPDRS III Off | LEDD |
| --- | --- | --- | --- | --- | --- | --- | --- | --- |
| Ekman total | DRT-PD  STN-DBS-PD | τb = .060  p = 0.725  τb = -.122  p = 0.491 | τb = -.127  p = 0.460  τb = -.242  p = 0.170 | τb = .048  p = 0.778  **τb = -.401**  **p = 0.022** | τb = -.172  p = 0.321  **τb = -.397**  **p = 0.033** | τb = .011  p = 0.947  τb = -.046  p = 0.809 | τb = .143  p = 0.399  τb = -.165  p = 0.287 | τb = .107  p = 0.527  τb = -.027  p = 0.879 |
| Ekman sadness | DRT-PD  STN-DBS-PD | τb = .071  p = 0.692  τb = -.172  p = 0.350 | τb = .032  p = 0.857  τb = .043  p = 0.816 | τb = .045  p = 0.801  τb = -.007  p = 0.969 | τb = .026  p = 0.885  τb = .141  p = 0.468 | τb = .097  p = 0.588  **τb = -.401**  **p = 0.041** | τb = .020  p = 0.914  τb = .046  p = 0.816 | τb = .070  p = 0.693  τb = .085  p = 0.642 |
| Ekman happiness | DRT-PD  STN-DBS-PD | τb = -.058  p = 0.766  τb = .010  p = 0.961 | τb = -.250  p = 0.197  τb = -.378  p = 0.058 | τb = -.210  p = 0.275  τb = -.366  p = 0.065 | τb = .078  p = 0.690  **τb = -.417**  **p = 0.047** | τb = -.082  p = 0.670  τb = .295  p = 0.167 | τb = .151  p = 0.436  τb = -.369  p = 0.084 | τb = .142  p = 0.458  τb = -.077  p = 0.697 |
| Ekman anger | DRT-PD  STN-DBS-PD | τb = -.045  p = 0.802  τb = -.222  p = 0.237 | τb = -.337  p = 0.057  τb = -.359  p = 0.054 | τb = -.032  p = 0.858  τb = -.197  p = 0.289 | τb = -.117  p = 0.516  τb = -.283  p = 0.153 | τb = -.165  p = 0.349  τb = -.066  p = 0.740 | τb = .287  p = 0.101  **τb = -.420**  **p = 0.036** | τb = .169  p = 0.334  τb = -.132  p = 0.479 |
| Ekman disgust | DRT-PD  STN-DBS-PD | τb = -.083  p = 0.641  τb = .051  p = 0.784 | τb = -.147  p = 0.409  τb = -.101  p = 0.585 | τb = -.082  p = 0.641  τb = -.186  p = 0.311 | τb = -.254  p = 0.159  τb = -.272  p = 0.160 | τb = .209  p = 0.240  τb = .203  p = 0.303 | τb = .019  p = 0.914  τb = .100  p = 0.612 | τb = -.246  p = 0.163  τb = -.036  p = 0.845 |
| Ekman fear | DRT-PD  STN-DBS-PD | τb = -.052  p = 0.773  τb = -.194  p = 0.281 | τb = -.065  p = 0.718  τb = -.275  p = 0.125 | τb = .045  p = 0.801  τb = -.314  p = 0.078 | τb = -.126  p = 0.490  τb = -.320  p = 0.084 | τb = -.037  p = 0.839  τb = -.124  p = 0.517 | τb = -.033  p = 0.856  τb = -.062  p = 0.749 | τb = .098  p = 0.585  τb = -.075  p = 0.673 |
| Ekman surprise | DRT-PD  STN-DBS-PD | τb = .276  p = 0.127  τb = -.168  p = 0.367 | τb = .249  p = 0.168  **τb = -.391**  **p = 0.035** | τb = .189  p = 0.293  **τb = -.432**  **p = 0.019** | τb = .194  p = 0.290  τb = -.250  p = 0.199 | τb = -.031  p = 0.865  τb = -.106  p = 0.592 | τb = -.241  p = 0.181  τb = -.239  p = 0.227 | τb = .204  p = 0.259  τb = .108  p = 0.557 |
| PACI CInt | DRT-PD  STN-DBS-PD | **τb = -.361**  **p = 0.044**  τb = -.163  p = 0.373 | **τb = -.559**  **p = 0.002**  **τb = -.506**  **p = 0.005** | **τb = -.517**  **p = 0.004**  τb = -.209  p = 0.247 | τb = -.138  p = 0.447  τb = -.147  p = 0.445 | τb = -.084  p = 0.683  τb = -.024  p = 0.903 | **τb = .434**  **p = 0.014**  τb = -.144  p = 0.462 | τb = .000  p = 1.000  τb = .112  p = 0.537 |
| PACI PhC | DRT-PD  STN-DBS-PD | τb = -.209  p = 0.245  τb = -.307  p = 0.099 | **τb = -.417**  **p = 0.020**  τb = -.326  p = 0.078 | τb = -.240  p = 0.179  τb = -.259  p = 0.159 | τb = -.113  p = 0.534  τb = -.203  p = 0.300 | τb = -.139  p = 0.436  τb = -.049  p = 0.804 | **τb = .455**  **p = 0.011**  τb = .046  p = 0.816 | τb = .101  p = 0.566  τb = .079  p = 0.667 |
| RME | DRT-PD  STN-DBS-PD | τb = .074  p = 0.671  τb = -.278  p = 0.124 | τb = -.055  p = 0.750  **τb = -.440**  **p = 0.014** | τb = .183  p = 0.289  τb = -.205  p = 0.250 | τb = .100  p = 0.569  τb = -.134  p = 0.475 | τb = -.120  p = 0.487  τb = -.186  p = 0.331 | τb = .146  p = 0.396  τb = -.071  p = 0.713 | τb = .187  p = 0.274  τb = .062  p = 0.730 |
| RME control | DRT-PD  STN-DBS-PD | τb = -.172  p = 0.344  τb = -.111  p = 0.554 | τb = -.258  p = 0.156  τb = .279  p = 0.135 | τb = -.059  p = 0.744  τb = -.007  p = 0.969 | τb = -.230  p = 0.214  τb = .137  p = 0.488 | τb = .247  p = 0.174  τb = -.306  p = 0.125 | τb = .046  p = 0.799  τb = .113  p = 0.573 | τb = -.216  p = 0.231  τb = .029  p = 0.875 |

BDI = Beck Depression Inventory; CInt = Communicative Intention; DRT-PD = patients with PD receiving dopaminergic replacement therapy; LEDD = L-dopa equivalent daily dose; PACI = Protocol for the Attribution of Communicative Intentions; PD = Parkinson’s disease; PhC = Physical causality; RME = Reading the Mind in the Eyes Test; STAI = State Trait Anxiety Inventory; STN-DBS-PD = patients with PD who were treated with deep brain stimulation of the subthalamic nucleus (STN-DBS); UPDRS = Unified Parkinson’s Disease Rating Scale.

**Table 6. Correlations between social cognitive and neuropsychological measures in DRT-PD.**

|  | Ekman  total | Ekman sadness | Ekman  happiness | Ekman  anger | Ekman  disgust | Ekman  fear | Ekman  surprise | PACI CInt | PACI PhC | RME | RME control |
| --- | --- | --- | --- | --- | --- | --- | --- | --- | --- | --- | --- |
| Corsi’s  Block-Tapping Test | τb = .246  p = 0.154 | τb = .148  p = 0.414 | τb = .047  p = 0.812 | τb = .000  p = 1.000 | τb = .347  p = 0.054 | τb = -.025  p = 0.891 | τb = .081  p = 0.657 | τb = -.049  p = 0.787 | τb = .123  p = 0.496 | τb = .075  p = 0.665 | τb = .282  p = 0.124 |
| Attentional matrices | **τb = .366**  **p = 0.027** | τb = .110  p = 0.524 | τb = .254  p = 0.174 | **τb = .383**  **p = 0.024** | τb = .326  p = 0.058 | τb = -.006  p = 0.973 | τb = .229  p = 0.190 | τb =.154  p = 0.369 | **τb = .473**  **p = 0.006** | **τb = .480**  **p = 0.004** | τb = .230  p = 0.190 |
| Raven’s Coloured Progressive Matrices | **τb = .350**  **p = 0.036** | τb = .082  p = 0.638 | τb = .036  p = 0.851 | **τb = .383**  **p = 0.026** | τb = .058  p = 0.739 | τb = .243  p = 0.167 | τb = .113  p = 0.522 | τb = .232  p = 0.182 | τb = .229  p = 0.190 | τb = .271  p = 0.108 | τb = .270  p = 0.129 |
| Bisyllabic word repetition test | τb = .272  p = 0.121 | τb = .076  p = 0.678 | τb = -.010  p = 0.961 | τb = .347  p = 0.055 | τb = .019  p = 0.918 | τb = .096  p = 0.603 | τb = .232  p = 0.212 | τb = -.025  p = 0.891 | τb = .051  p = 0.782 | **τb = -.486**  **p = 0.006** | τb = -.006  p = 0.972 |
| Paired-associate  learning | τb = .060  p = 0.725 | τb = -.039  p = 0.827 | τb = -.097  p = 0.619 | **τb = .385**  **p = 0.029** | τb = -.104  p = 0.564 | τb = -.046  p = 0.799 | τb = .046  p = 0.800 | τb = .091  p = 0.614 | τb = .085  p = 0.636 | **τb = .416**  **p = 0.016** | τb = .000  p = 1.000 |
| Trail Making Test  Part B | **τb = -.376**  **p = 0.023** | τb = .006  p = 0.973 | τb = -.219  p = 0.241 | **τb = -.405**  **p = 0.017** | τb = -.189  p = 0.273 | τb = -.262  p = 0.131 | τb = .006  p = 0.973 | **τb = -.337**  **p = 0.050** | **τb = -.421**  **p = 0.015** | **τb = -.398**  **p = 0.017** | τb = -.271  p = 0.122 |
| NCST  categories | τb = .231  p = 0.236 | τb = .209  p = 0.308 | τb = .030  p = 0.892 | τb = .348  p = 0.084 | τb = -.010  p = 0.962 | τb = .260  p = 0.206 | τb = -.242  p = 0.243 | τb = .295  p = 0.148 | **τb = .446**  **p = 0.029** | τb = .206  p = 0.296 | τb = .446  p = 0.032 |
| NCST  errors | τb = -.084  p = 0.622 | τb = .114  p = 0.521 | τb = .291  p = 0.131 | **τb = -.345**  **p = 0.049** | τb = -.077  p = 0.663 | τb = .006  p = 0.973 | τb = .000  p = 1.000 | τb = .077  p = 0.663 | τb = -.138  p = 0.437 | τb = -.266  p = 0.121 | τb = .086  p = 0.635 |
| NCST  perseverations | τb = -.300  p = 0.087 | τb = -.044  p = 0.810 | τb = .220  p = 0.269 | τb = -.376  p = 0.038 | τb = -.113  p = 0.539 | τb = -.153  p = 0.408 | τb = -.186  p = 0.317 | τb = -.050  p = 0.785 | τb = -.215  p = 0.242 | **τb = -.370**  **p = 0.037** | τb = -.136  p = 0.468 |
| Phonemic verbal  fluency | τb = .114  p = 0.493 | **τb = .356**  **p = 0.041** | τb = -.212  p = 0.260 | τb = .301  p = 0.079 | τb = -.012  p = 0.947 | τb = -.047  p = 0.788 | τb = .083  p = 0.638 | τb = .006  p = 0.973 | τb = .047  p = 0.789 | **τb = .330**  **p = 0.049** | τb = .065  p = 0.711 |
| Category verbal  fluency | τb = .060  p = 0.725 | τb = .300  p = 0.095 | τb = -.019  p = 0.921 | τb = .188  p = 0.285 | τb = -.077  p = 0.667 | τb = -.092  p = 0.610 | τb = .144  p = 0.425 | τb = .038  p = 0.830 | τb = -.038  p = 0.829 | τb = .261  p = 0.129 | τb = .158  p = 0.383 |

CInt = Communicative Intention; DRT-PD = patients with PD receiving dopaminergic replacement therapy; NCST = Nelson Modified Card Sorting Test; PACI = Protocol for the Attribution of Communicative Intentions; PD = Parkinson’s disease; PhC = Physical causality; RME = Reading the Mind in the Eyes Test.

**Table 7. Correlations between social cognitive and neuropsychological measures in STN-DBS-PD.**

|  | Ekman  total | Ekman sadness | Ekman  happiness | Ekman  anger | Ekman  disgust | Ekman  fear | Ekman  surprise | PACI CInt | PACI PhC | RME | RME control |
| --- | --- | --- | --- | --- | --- | --- | --- | --- | --- | --- | --- |
| Corsi’s  Block-Tapping Test | τb = -.057  p = 0.757 | τb = .202  p = 0.287 | **τb = -.448**  **p = 0.030** | τb = -.054  p = 0.780 | τb = -.121  p = 0.526 | τb = -.058  p = 0.755 | τb = -.160  p = 0.404 | τb = -.015  p = 0.938 | τb = .099  p = 0.605 | τb = -.065  p = 0.726 | **τb = .408**  **p = 0.034** |
| Attentional matrices | **τb = .500**  **p = 0.004** | τb = .084  p = 0.642 | **τb = .432**  **p = 0.029** | τb = .174  p = 0.346 | τb = .292  p = 0.110 | **τb = .402**  **p = 0.024** | **τb = .581**  **p = 0.002** | τb =.174  p = 0.335 | τb = .308  p = 0.093 | **τb = .443**  **p = 0.013** | τb = -.058  p = 0.753 |
| Raven’s Coloured Progressive Matrices | τb = -.014  p = 0.939 | τb = .209  p = 0.259 | τb = -.295  p = 0.143 | τb = -.089  p = 0.636 | τb = -.168  p = 0.368 | τb = .056  p = 0.758 | τb = -.015  p = 0.937 | τb = .150  p = 0.416 | τb = .015  p = 0.937 | τb = -.077  p = 0.672 | τb = .007  p = 0.968 |
| Bisyllabic word repetition test | **τb = .368**  **p = 0.044** | τb = -.037  p = 0.844 | τb = .193  p = 0.348 | τb = .162  p = 0.402 | **τb = .491**  **p = 0.010** | τb = .274  p = 0.139 | τb = .183  p = 0.340 | τb = .236  p = 0.209 | **τb = .594**  **p = 0.002** | τb = .347  p = 0.061 | τb = .100  p = 0.603 |
| Paired-associate  learning | τb = -.171  p = 0.358 | τb = .188  p = 0.328 | τb = -.164  p = 0.433 | τb = -.355  p = 0.070 | τb = .049  p = 0.798 | τb = -.151  p = 0.424 | τb = -.183  p = 0.346 | τb = .202  p = 0.291 | τb = -.008  p = 0.966 | τb = .040  p = 0.833 | τb = .067  p = 0.731 |
| Trail Making Test  Part B | τb = -.020  p = 0.909 | τb = .028  p = 0.877 | τb = .163  p = 0.409 | τb = -.160  p = 0.388 | τb = .021  p = 0.907 | τb = .048  p = 0.789 | τb = -.093  p = 0.611 | τb = -.021  p = 0.908 | τb = -.179  p = 0.328 | τb = -.293  p = 0.100 | τb = .015  p = 0.937 |
| NCST  categories | τb = .262  p = 0.204 | τb = -.031  p = 0.886 | **τb = .524**  **p = 0.026** | τb = .286  p = 0.192 | τb = .016  p = 0.943 | τb = .134  p = 0.524 | τb = .298  p = 0.171 | τb = .350  p = 0.101 | τb = -.016  p = 0.942 | τb = -.194  p = 0.357 | τb = -.064  p = 0.772 |
| NCST  errors | **τb = -.523**  **p = 0.004** | τb = -.154  p = 0.411 | τb = -.181  p = 0.377 | τb = -.235  p = 0.219 | τb = -.305  p = 0.106 | **τb = .436**  **p = 0.012** | **τb = -.495**  **p = 0.009** | **τb = -.429**  **p = 0.022** | **τb = -.450**  **p = 0.018** | **τb = -.420**  **p = 0.022** | τb = -.137  p = 0.475 |
| NCST  perseverations | τb = -.148  p = 0.431 | τb = -.086  p = 0.659 | τb = .096  p = 0.652 | τb = .137  p = 0.490 | τb = -.016  p = 0.936 | τb = -.310  p = 0.104 | τb = -.215  p = 0.275 | τb = -.348  p = 0.073 | τb = -.104  p = 0.599 | τb = -.189  p = 0.322 | τb = -.121  p = 0.542 |
| Phonemic verbal  fluency | **τb = .628**  **p < 0.001** | τb = .288  p = 0.119 | **τb = .413**  **p = 0.040** | τb = -.297  p = 0.115 | τb = .328  p = 0.078 | **τb = .509**  **p = 0.005** | **τb = .565**  **p = 0.002** | τb = .221  p = 0.229 | τb = .301  p = 0.107 | **τb = .481**  **p = 0.008** | τb = -.074  p = 0.693 |
| Category verbal  fluency | τb = .330  p = 0.062 | τb = -.007  p = 0.969 | τb = .126  p = 0.527 | τb = .235  p = 0.208 | τb = .331  p = 0.072 | τb = .179  p = 0.318 | τb = .254  p = 0.170 | τb = .267  p = 0.142 | τb = .275  p = 0.136 | τb = .248  p = 0.167 | τb = -.271  p = 0.145 |

CInt = Communicative Intention; NCST = Nelson Modified Card Sorting Test; PACI = Protocol for the Attribution of Communicative Intentions; PD = Parkinson’s disease; PhC = Physical causality; RME = Reading the Mind in the Eyes Test; STN-DBS-PD = patients with PD who were treated with deep brain stimulation of the subthalamic nucleus (STN-DBS).
